# Effects of L-cysteine on the synthesis and secretion of extracellular pigments in submerged fermentation of *Monascus purpureus* S109

**DOI:** 10.3389/fmicb.2026.1777888

**Published:** 2026-04-01

**Authors:** Sixu Lin, Xue Yang, Junyao Wang, Qinghua Yu, Bing Li, Xuerui Yan

**Affiliations:** College of Food Science, Shenyang Agricultural University, Shenyang, China

**Keywords:** extracellular *Monascus* pigments, L-cysteine, *Monascus* purpureus, submerged fermentation, synthesis and secretion

## Abstract

Extracellular *Monascus* pigments (eMPs), as a crucial component of *Monascus* pigments (MPs) with functional activities, have been widely utilized. In this study, the effect of L-cysteine on the eMPs production capacity of S109 were investigated, along with the physiological and molecular response of S109 under L-cysteine. The results indicated that increasing the concentration of L-cysteine could promote the growth of S109 and improve the cell membrane permeability and fluidity. However, high concentration of L-cysteine did not constantly promote the yield of eMPs. The optimal condition was achieved with the addition of 2 g/L L-cysteine, under which the production of eMPs increased from 80.42 ± 0.37 to 329.20 ± 2.69 U/mL. Under this condition, all genes involved in the MPs synthesis and secretion pathway were approximately up-regulated. This study proposed a simple and effective method to improve the eMPs, which laid the foundation for the substantial improvement of *Monascus* fermentation industry.

## 1 Introduction

MPs are secondary metabolites produced by *Monascus* spp. and are predominantly accumulated within the mycelia. The intracellular pigments (iMPs) can only be released through cell disruption, which consequently increases both the production cost and the technical complexity of MPs manufacturing (Liu et al., 2019a). In terms of functionality and stability, eMPs exhibit better water solubility than iMPs and can serve as natural alternatives to synthetic dyes (Ferysiuk and Wojciak, 2020). eMPs exhibit antioxidant and antibacterial activities and can be readily utilized, whereas iMPs are confined within the mycelium, and their bioactive components (antioxidant and cholesterol-lowering) are difficult to exert directly before being released (Farawahida et al., 2022; Husakova et al., 2024). In addition, eMPs are less sensitive to pH, and their photostability is higher than that of iMPs under identical light conditions (Bai et al., 2022; Yin et al., 2022b). Submerged fermentation is the main method for industrial production of MPs (Liu et al., 2019a). However, during submerged fermentation, the iMPs contents are higher, resulting in a relatively low MPs in the fermentation medium and consequently increasing downstream processing costs. Therefore, enhancing the yield and productivity of eMPs is of significant practical importance for industrial-scale production.

Amino acids, as a source of nitrogen, is not only an essential nutrient for the growth of *Monascus* mycelium, but also plays a role in regulating the synthesis of metabolites. The 20 amino acids were screened by Yin et al. (2022b), and found that histidine had the most significant effect on the MPs of *Monascus purpureus* RP2, and its MPs content reached 200 U/mL. This was attributed to the histidine enhanced transcriptions of most of the PKS cluster genes, especially the expression levels of 6 key enzymes (PKS Mon2A4603, short-chain alcohol dehydrogenase Mon2A4601, 3-O-acetyltransferase Mon2A4600, oxidoreductase Mon2A4599, FAD-dependent dehydrogenase Mon2A4598, and serine hydrolase Mon2A4597) encoding genes were significantly increased as MPs biosynthesis enhanced. Arginine promoted the MPs synthesis of *Monascus purpureus* M1, and its content was 245 U/mL (Zhang et al., 2022). However, the addition of specific bioactive amino acids such as methionine can significantly inhibit the synthesis of MPs in *Monascus purpureus* RP2 and reduce the pigment yield by 60–70% (Yin et al., 2022a). This was because methionine could inhibit the production of MPs by inducing the expression of s-adenosylmethionine synthase and the synthesis of s-adenosylmethionine. Therefore, different nitrogen sources exerted distinct regulatory effects on the biosynthesis of MPs. In advance, it was identified that L-cysteine notably enhanced MPs synthesis, particularly promoting the production of eMPs. L-cysteine is not only extensively applied as a food additive in the food industry but also plays an important role in antioxidation, facilitation of protein synthesis, and metabolic regulation. Research has indicated that L-cysteine can participate in redox reactions to help cells counteract oxidative stress, thereby protecting them from damage (Park et al., 2024). L-cysteine can also produce glutathione, thereby enhancing the antioxidant capacity of cells (Huang et al., 2017a).

Although several studies have investigated factors influencing MPs production, the mechanism of L-cysteine in eMPs synthesis and secretion during submerged fermentation of *Monascus purpureus* (*M. purpureus*) remains largely unexplored. In particular, the effects of L-cysteine on the physiological state, membrane properties, and the mechanisms governing eMPs synthesis and transport in *M. purpureus* S109 have not been elucidated. To address this gap, this study proposed a new strategy for enhancing eMPs production in S109 by adding L-cysteine under submerged fermentation. By analyzing key fermentation parameters (mycelial biomass, residual sugar, eMP and citrinin content), membrane properties, NAD^+^/NADH levels, and the expression of genes involved in eMPs synthesis and secretion, this study elucidated the potential mechanism through which L-cysteine regulates eMPs biosynthesis and secretion.

## 2 Materials and methods

### 2.1 Strain source and fermentation conditions

*M. purpureus* strain S109 was isolated from Hong Qu rice and is preserved in Food Science College (Shenyang Agricultural University, China). This wild *M. purpureus* strain with high yield of MPs and no citrinin.

During the initial growth phase, *M.purpureus* S109 was inoculated on PDA medium and cultured at 30°C for 7 days. Subsequently, the mycelia were rinsed with sterile water to prepare a spore suspension at a concentration of 1 × 10^6^ spores/mL. An 8% (v/v) spore suspension was then inoculated into the 50 mL seed medium and cultured at 30 °C under constant shaking for 96 h to obtain the seed liquid.

A 20% (v/v) seed liquid was inoculated into 250 mL Erlenmeyer flasks containing 50 mL of submerged fermentation medium for batch fermentation. No additional feeding was applied during the cultivation process. The flasks were incubated at 30 °C in a temperature-controlled rotary shaker (TY-DY3, Tianyan) at 180 rpm for 2–14 days. After fermentation, the entire culture broth from each flask was collected and subjected to vacuum filtration. The filtrate was used for the determination of eMPs content and residual sugar concentration. The retained mycelium was used for iMPs extraction. After extraction, the mixture was filtered again, and the supernatant was collected for iMPs quantification. The remaining mycelial residue was dried to constant weight for biomass determination.

Both the seed medium and the submerged fermentation medium were composed of 5% glucose, 1.5% peptone, 0.2% NaNO_3_, 0.1% MgSO_4_⋅7H_2_O, and 0.15% KH_2_PO_4_, with the pH adjusted to 5.5 using lactic acid, and each medium was sterilized at 121 °C for 30 min.

### 2.2 Addition of L-cysteine

Based on previous study (Chen G. et al., 2017; Yin et al., 2022b) and preliminary experiments, the effects of different concentrations of L-cysteine on the growth, cell membrane changes and eMPs of S109 strain were investigated. with 0 g/L L-cysteine serving as the control group, the low (2 g/L), medium (10 g/L), and high (50 g/L) concentrations L-cysteine was added to the submerged fermentation broth containing 20% (v/v) S109 seed liquid, respectively, and cultured at 30°C under constant shaking for 14 days (this fermentation days could complete the main metabolic process) (Wang et al., 2020). Then, the concentration of L-cysteine was carefully screened to determine the optimal addition amount. The method was the same as above.

### 2.3 Determination of mycelial biomass, residual sugar, and pH

The mycelium was obtained by centrifugation, and washed with distilled water and filtered using filter paper that had been pre-dried to constant weight (with a mass of m_1_). Subsequently, the filter paper along with the mycelia was dried at 50 °C until a constant weight was achieved, and the combined mass of the filter paper and myceliumt was recorded as m_2_. The mass was measured using an analytical balance (PX224ZH/E, OHAUS). The mycelial biomass (A) was then calculated using the formula:


A(g⋅L)-1=(m-2m)1×100

The residual sugar content in the fermentation broth was determined using the 3,5-dinitrosalicylic acid (DNS) method (Zhang Y. et al., 2024). Briefly, 1.5 mL of the fermentation broth was centrifuged at 8,000 r/min for 5 min to obtain the supernatant. Then, 1 mL of the supernatant was mixed with 1 mL of DNS reagent. After thorough mixing, the solution was heated in a boiling water bath at 100 °C for 5 min to develop the color. After cooling, the sample was diluted with 6 mL of distilled water, and the absorbance at 540 nm was measured by UV-visible spectrophotometer (UV-2700). The residual sugar content in the fermentation broth was calculated according to the following equation:


R⁢e⁢s⁢i⁢d⁢u⁢a⁢l⁢s⁢u⁢g⁢a⁢r⁢c⁢o⁢n⁢c⁢e⁢n⁢t⁢r⁢a⁢t⁢i⁢o⁢n⁢(g/L)=(M/V)×n

Where M is the calculated reducing sugar content (mg), V is the DNS reagent added volume (mL), n is the dilution multiple.

The pH value of the fermentation broth was measured using a pH meter (PH5S-E).

### 2.4 Determination of MPs

Following the method described by Liu H. et al. (2021) with minor modifications, the fermentation broth containing different concentrations of L-cysteine was vacuum-filtered to obtain the supernatant. A 2.5 mL aliquot of the supernatant was transferred to a test tube and diluted with 5 mL of distilled water. The absorbance was measured using a UV-visible spectrophotometer (UV-2700). The absorbance readings at 410, 470, and 510 nm were multiplied by the corresponding dilution multiple to obtain the contents of yellow, orange, and red eMPs, respectively. The total eMPs content was the sum of the three.

Similarly, the mycelia recovered from the vacuum filtration were washed with distilled water and then subjected to extraction by adding 50 mL of 70% ethanol solution followed by incubation in a water bath at 60 °C for 1 h. After filtration through filter paper, a 2.5 mL aliquot of the resulting supernatant was diluted with 5 mL of 70% ethanol solution. The absorbance values at 410 nm, 470 nm and 510 nm were measured by UV-visible spectrophotometer (UV-2700), and multiplied by the dilution multiple to obtain the contents of yellow iMPs, orange iMPs and red iMPs, respectively. The total iMPs content was the sum of the three.

### 2.5 Determination of citrinin content

For each sample, 0.5 mL of fermentation broth with varying concentrations of L-cysteine was mixed with 1 mL of 75% methanol. The mixture was then subjected to extraction in a water bath at 60 °C for 1 h. After cooling to 25 °C, the sample was centrifuged at 8,000 rpm for 5 min, and the supernatant was obtained by filtration through a 0.22 μm organic membrane. The content of citrinin in samples was determined by Shimadzu high performance liquid chromatography with Photodiode Array (PDA) detector and Inert Systain AQ-C18 column (250 × 4.6 mm, 5 μm). The column temperature was 30°C, the flow rate was 1 mL/min, the mobile phase was methanol: phosphoric acid aqueous solution (pH = 3.0) = 55:45 (v/v), and the PDA wavelength was 210 nm.

### 2.6 Cell membrane fatty acid composition analysis

Fermentation broths containing different concentrations of L-cysteine were centrifuged at 8,000 rpm for 5 min to collect the mycelia. The supernatant was discarded and the collected mycelia were washed three times with deionized water. The cell membrane fatty acids were then purified and methylated following the method described by Chen G. et al. (2017). The fatty acid composition was subsequently analyzed using GC-MS method, as detailed by Huang et al. (2017b).

### 2.7 Determination of cell membrane permeability

An 1 mL conventional fermentation broth (CFB) without L-cysteine was diluted 100-fold with deionized water to adjust the absorbance of the mycelial suspension to 0.20–0.25 at 600 nm. Then, 1.5 mL of the mycelial suspension was mixed with 0.5 mL of L-cysteine aqueous solution, and the mixture was incubated for 10 min. Deionized water served as the blank control, with an additional reference blank prepared by mixing 1.5 mL of the mycelial suspension with 0.5 mL of deionized water. After centrifugation, 1.0 mL of the supernatant was collected and diluted with 4.0 mL of deionized water to determine the change of conductivity.

Tyrosine and tryptophan residues in cell membrane proteins have certain fluorescence, and the fluorescence intensity can directly reflect the change of cell membrane permeability (Tiwari et al., 2023). Following the above method to obtain the 0.20–0.25 OD_600_ mycelial suspension. 1.5 mL of the mycelial suspension was mixed with 0.5 mL of L-cysteine aqueous solution and incubated for 10 min. After centrifugation at 10,000 rpm for 5 min, the fluorescence intensity of the supernatant was measured. Tyrosine and tryptophan were excited at wavelengths of 297 and 292 nm, respectively, with both emission wavelengths set at 348 nm.

### 2.8 Determination of cell membrane potential

The cell membrane potential was determined using the Rhodamine 123 (Rh123) assay (Zorova et al., 2022). The CFB was centrifuged to collect the mycelia, which were then washed three times with deionized water. The mycelia were subsequently resuspended in 0.1 M phosphate-buffered saline (PBS), and the resulting cell suspension was diluted 10-fold. Next, 1 mL of the diluted cell suspension was mixed with 1.5 mL of L-cysteine a queous solution and incubated at 30 °C under constant shaking for 30 min. After adding 10 μL of a 1 g/L Rh123 solution, the mixture was incubated in the dark for 10 min, followed by centrifugation at 8,000 rpm for 5 min. The supernatant was discarded, and the mycelia were washed three times with 0.1 M PBS and resuspended. The fluorescence intensity of Rh123 in the mycelium was measured at an excitation wavelength of 488 nm and an emission wavelength of 530 nm.

### 2.9 Determination of K^+^ and Na^+^ concentration

To evaluate the secretion of intracellular KK^+^ and NaK^+^, 20 mL of the CFB as supplemented with L-cysteine at concentrations of 2, 10, or 50 g/L, respectively. The cultures were incubated at 30 °C with shaking at 180 rpm for 1 h, then centrifuged at 8,000 rpm for 5 min. The supernatant was collected by filtration, and the concentrations of KK^+^ and NaK^+^ were measured using Flame Atomic Absorption Spectrophotometer, following the method described by Chen G. et al. (2017).

### 2.10 Determination of NAD^+^/NADH

From each treatment group with different L-cysteine concentrations, 0.1 g of mycelia was collected to determine the intracellular levels of NAD^+^ and NADH. The NAD^+^/NADH contents were measured using the NAD^+^/NADH assay kit (Solabel Technology Co., Beijing, China) according to the manufacturer’s instructions. The concentrations were determined at 570 nm.

### 2.11 Comparative analysis of sulfur source and thiol group

To investigate the effects of different sulfur-containing amino acids on the eMPs production of S109. Taking CFB as the control group, 2 g/L L-methionine, 2 g/L L-cysteine, 2 g/L L-cystine, and 2 g/L D-cysteine were added to the submerged fermentation medium containing 20% (v/v) S109 seed solution, respectively. After 12 days of constant temperature shaking culture at 30°C, the eMPs was measured. The aim was to verify whether the promoting effect of L-cysteine on eMPs was attributed to the supply of sulfur or the specific action of thiol group.

### 2.12 *RT-qPCR* analysis

Real-time quantitative PCR (*RT-qPCR*) was employed, following the method described by Zhen et al. (2019) to measure L-cysteine addition on the expression of key genes involved in the biosynthesis and secretion of MPs. Mycelia treated with different concentrations of L-cysteine were collected and rapidly ground into a fine powder in liquid nitrogen. Total RNA was extracted using Trizol reagent (Sangon Biotech, Shanghai, China), and cDNA was synthesized using the HiFiScript cDNA Synthesis Kit (CWBiotech, Beijing, China). *RT-qPCR* amplification and analysis were performed using the UltraSYBR Mixture (Low ROX) kit (CWBiotech, Beijing, China). The 20 μL reaction system comprised 10 μL of UltraSYBR Mixture, 0.4 μL each of forward and reverse primers (10 μmol/L), 2 μL of cDNA, and 7.2 μL of ddH2O, with actin serving as the internal reference gene (Qian et al., 2021). The relative genes expression were calculated using the 2^–ΔΔ*CT*^ method. The primer sequences were shown in Table 1, which were designed based on the cDNA sequences of *MrpigA* (GenBank accession No. ALN44200.1), *MrpigB* (AGL44390.1), *MrpigC* (ALN44201.1), *MrpigD* (AGI63864.1), *MrpigE* (AHA93896.1), *MrpigF* (KX278306), *MrpigG* (KX278307), *MrpigH* (KX278308), *MrpigI* (KX278309), *MrpigJ* (JX675042), *MrpigK* (JX675043), *MrpigL* (KX290843), *MrpigM* (KX278310), *MrpigN* (ALT31754.1), *MrpigO* (KX290844), and *MrpigP* (KX290845) (Chen W. et al., 2017).

**TABLE 1:** Primers used in this study.

Gene	Sense primer	Anti-sense primer
Actin	CAGGGAGAAGATGACCCAGA	GTCACCAGAGTCAAGCACGA
*MrpigA*	GTCATTGGCATGTCGTGTAAGG	GCATCGTGGTCTCGGATAAAG
*MrpigB*	GATTGACGGAGCAAACAGCC	CTGCGTGATGGTTTCTGATAGG
*MrpigC*	AGCCAATGTGCCATGGACAGGA	TCCTCCGGTGATGAAGACTGCT
*MrpigD*	AACACTGGGCTATTCAGGAGCG	TGCAATGATGCTGCCGTCCA
*MrpigE*	AGCAAGTACACCAGGTACATACGC	TGGTTACCGTTGCTCCTTCGAAT
*MrpigF*	ACCCATCTCTATTCAGCGAGAATG	CGGAGGATTTCTCGACCAGAGT
*MrpigG*	TCCTCACGCGGTACTTGTCCA	TGGATCGATCTCGGGATGGT
*MrpigH*	AACGTCACTCGCTTCCAGGT	AGTTGGTCCTGCTGGAAGAG
*MrpigI*	GTCCAGACGCCGGTGCAGTA	GGAGCGGGAGTAGGCATAACG
*MrpigJ*	CGCCCGTAAAGTTCGTCAGTA	GCGTGGTGAATCGGTAGACA
*MrpigK*	GAGGAAATGTCTACCACCGAGG	GAATCCACCGACCAACCAGT
*MrpigL*	CTTCAGGGCATCTTTCAGT	GGAGTTTCTGGCACTCTTT
*MrpigM*	ATCCTTCAAACCGCTCAACCG	CGGACACTTTGTACAGTTTCGCAA
*MrpigN*	GTCACGAAAGAAGAGCCACAGCT	TCCAATTCCGGCTCCGATTTCG
*MrpigO*	TATTGGAGATCGTGCTCATCCACT	TGTGCAAAGCGCATCGTCTT
*MrpigP*	GTTGTCATCGGGCTCTTC	CCACTCAGCATCGCATT

### 2.13 Statistical analysis

All experiments were performed in triplicate at a minimum, and the data are expressed as the mean ± standard deviation (SD). Statistical analysis was conducted using SPSS 22.0 software. A one-way analysis of variance (ANOVA) was used for data analysis. Tukey’s multiple comparison test to assess the significance of differences, with *P* < 0.05, *P* < 0.01 considered statistically significant.

## 3 Results

### 3.1 Effect of L-cysteine on the growth and MPs content of S109

**FIGURE 1:**
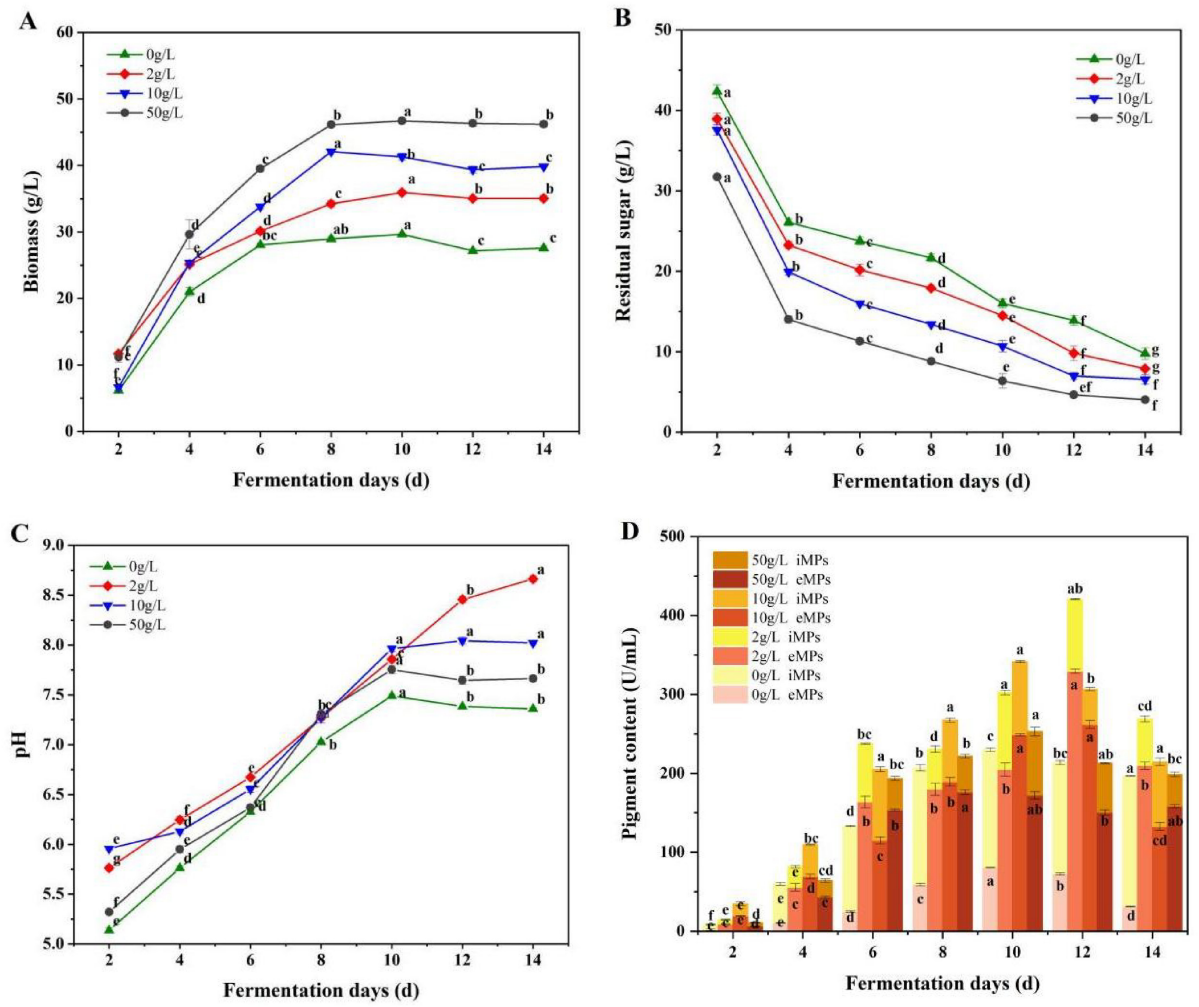
Effects of different L-cysteine concentrations on the growth and the MPs content of *M.purpureus* S109. **(A)** Biomass, **(B)** Residual sugar, **(C)** pH, **(D)** eMPs, iMPs and total MPs content. Data were presented as mean ± SD (*n* = 3). Different letters indicated significant differences among groups according to one-way ANOVA followed by Tukey’s multiple comparison test (*P* < 0.05).

The effects of different L-cysteine concentrations on the growth of strain S109 during fermentation were shown in Figure 1. Compared to CFB, the mycelial biomass of S109 under L-cysteine supplementation was markedly improved (Figure 1A). The biomass of high L-cysteine concentration (50 g/L) increased more significantly, with the dry weight of mycelia reaching 46.33 ± 0.49 g/L, which was 1.5-fold of the dry weight of mycelium of CFB (29.66 ± 0.06 g/L). After 8 days of fermentation with different L-cysteine concentrations, the mycelial biomass tended to be gentle.

L-cysteine also promoted the absorption of nutrients such as glucose by S109 (Figure 1B). Increasing the concentration of L-cysteine significantly enhanced the glucose utilization rate. After 12 days of fermentation with different L-cysteine concentrations, the residual sugar contents were lower. The concentration of exogenous additives was closely related to cell growth. High L-cysteine concentration promoted cell growth and significantly increased glucose utilization.

Furthermore, compared to CFB, L-cysteine exerted a notable influence on the fermentation environment of S109 (Figure 1C). L-cysteine increased the pH value by providing amino groups, thereby promoting the synthesis of red (Bai et al., 2022). At a low concentration of L-cysteine (2 g/L), the pH of the fermentation broth reached 8.6. However, as the concentration increased, a gradual decline in pH was observed.

The concentration of exogenous additives affected the metabolic synthesis of eMPs (Huang et al., 2017b). In the fermentation process, the effects of different concentrations of L-cysteine on eMPs and iMPs content were shown in Figure 1D. the iMPs content of S109 in CFB was 149.44 ± 2.21 U/mL, which was 1.8-fold that of eMPs content (80.42 ± 0.37 U/mL). After the addition of L-cysteine, the eMPs content was significantly increased. The eMPs content of S109 fermented by 2 g/L L-cysteine could reach 329.20 ± 2.69 U/mL, which was 3-fold of the iMPs content. With the increase of L-cysteine concentration, the eMPs content of S109 increased first and then decreased. The eMPs content at 50 g/L L-cysteine was 176.24 ± 2.85 U/mL, representing a significant reduction compared with that achieved at low concentrations (*P* < 0.05).

In summary, L-cysteine significantly promoted the growth of S109 and significantly increased the content of eMPs and total MPs. Among them, the content of eMPs (329.20 ± 2.69 U/mL) and total MPs (420.54 ± 2.37 U/mL) produced by 2 g/L L-cysteine fermentation after 12 days was significantly better than other concentrations (*P* < 0.05). Furthermore, the MPs yield of this study was 3. 74-, 1. 62-, and 5.57-fold higher than those reported by Huang et al. (2023), Yi et al. (2024), and He et al. (2022) respectively, and no citrinin was detected.

### 3.2 Effect of L-cysteine on fatty acid composition of cell membrane of S109

Fatty acid composition played a crucial role in the synthesis of MPs. The fatty acid composition of cell membrane was shown in Table 2. The highest content in different concentrations of L-cysteine and CFB was octadecenoic acid (18:1). The content of stearic acid (18:0) in CFB was significantly higher than that in L-cysteine (*P* < 0.05), while the content of palmitoleic acid (16:1) in L-cysteine was significantly higher than that in CFB (*P* < 0.05). Heptadecanoic acid (17:0) was only detected in CFB and 2 g/L L-cysteine.

**TABLE 2:** Fatty acid composition (% total fatty acid) in cell membranes of S109 in submerged fermentation without (CFB) and with addition of L-cysteine.

Fatty acid composition	L-cysteine concentration*			
	CFB	2 g/L	10 g/L	50 g/L
Saturated fatty acid				
Hexadecanoic acid (16:0)	1.49 ± 0.03	0.03 ± 0.01	0.88 ± 0.11	0.05 ± 0.02
Stearic acid (18:0)	25.73 ± 0.12	4.44 ± 0.31	2.21 ± 0.26	3.79 ± 0.47
Heptadecanoic acid (17:0)	11.68 ± 0.51	0.03 ± 0.01	–	–
Unsaturated fatty acid				
Palmitoleic acid (16:1)	2.72 ± 0.03	19.52 ± 0.09	19.46 ± 0.07	17.45 ± 0.04
Octadecenoic acid (18:1)	53.45 ± 0.74	72.81 ± 0.85	73.32 ± 0.41	74.42 ± 0.25
9 (Z),12 (Z)-Octadecadienoic acid (18:2)	4.93 ± 0.36	3.17 ± 0.93	4.13 ± 0.63	5.28 ± 0.54
Unsaturated/saturated fatty acids ratio^a^	1.58 ± 0.05	21.22 ± 1.01	31.36 ± 1.33	25.30 ± 1.54
IUFA (Index of unsaturated fatty acid)^b^	66.03 ± 1.07	98.67 ± 0.34	101.04 ± 0.5	102.43 ± 0.88

*CFB: Conventional fermentation for 12 days, 2, 10, 50 g/L were different concentrations of L-cysteine added to the fermentation broth, and fermentation for 12 days. Data are mean ± standard deviation (*n* = 3). Means in a row with different lowercase/capital letters are significantly different (*P* < 0.05). “–” indicated that the substance was not detected.

*^a^*(C16:1 + C18:1 + C18:2)/(C16:0 + C17:0 + C18:0).

*^b^*C16:1 + C18:1 + 2 × C18:2.

The degree of unsaturation of membrane lipids was expressed by the unsaturated/saturated fatty acids ratio (USFAR) and index of unsaturated fatty acid (IUFA), which represented the permeability and fluidity of the cell membrane (Wei et al., 2022). With the increase of L-cysteine concentration, the USFAR increased from 1.58 ± 0.05 to 21.22 ± 1.01, 31.36 ± 1.33 and 25.30 ± 1.54, respectively, and IUFA increased from 66.03 ± 1.07 to 98.67 ± 0.34, 101.04 ± 0.5 and 102.43 ± 0.88, respectively. L-cysteine significantly promoted the synthesis of unsaturated fatty acids, especially 2 g/L L-cysteine. High proportion of USFAR could modify cell membrane structure, improve its fluidity and permeability, leading to trans-membrane transformation and iMPs secretion (Wei et al., 2022).

### 3.3 Effects of L-cysteine on cell membrane function and NAD^+^/NADH of S109

**FIGURE 2:**
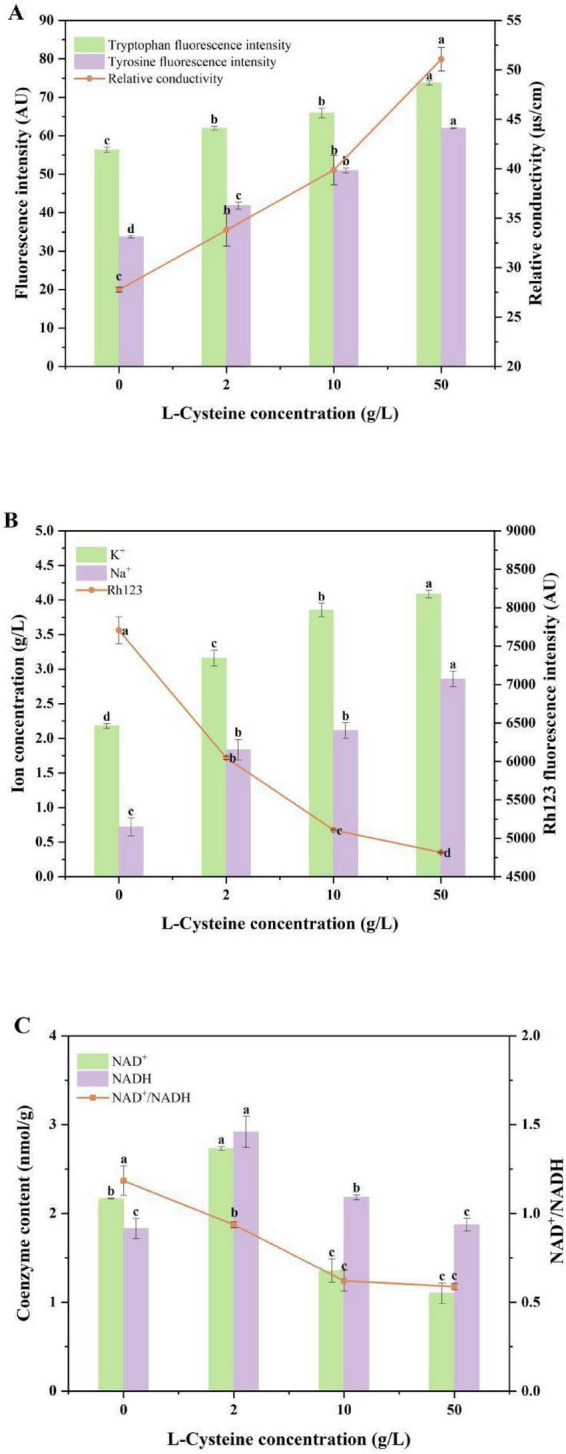
Effects of different L-cysteine concentrations on cell membrane permeability, membrane potential, ion concentrations and coenzyme content changes. **(A)**, Tryptophan, tyrosine fluorescence intensity and relative conductivity, **(B)**, Rh123 fluorescence intensity and K^+^, Na^+^ concentration, **(C)**, NAD^+^, NADH content and NAD^+^/NADH changes. Data were presented as mean ± SD (*n* = 3). Different letters indicated significant differences among groups according to one-way ANOVA followed by Tukey’s multiple comparison test (*P* < 0.05).

The impact of varying concentrations of L-cysteine on the membrane permeability of *M. purpureus* S109 was illustrated in Figure 2A. Relative conductivity in the fermentation broth increased significantly with rising L-cysteine concentrations compared with CFB (*P* < 0.05). Tryptophan and tyrosine residues on the cell membrane had fluorescence intensity, which reflected changes in membrane protein structure (Guo et al., 2020; Sathya et al., 2023). High L-cysteine concentration significantly increased the levels of tryptophan and tyrosine in the fermentation broth (*P* < 0.05).

Rh123 has certain fluorescence, and its fluorescence intensity in the cell can show the change of cell membrane potential. As shown in Figure 2B, compared with CFB, the fluorescence intensity of intracellular Rh123 decreased by 21.5% when 2 g/L L-cysteine was added, and increasing concentrations of L-cysteine resulted in a progressive decrease in Rh123 fluorescence intensity. Moreover, during fermentation with 2 g/L L-cysteine, the concentration of K^+^ and Na^+^ in the extracellular fluid increased significantly. As the concentration of L-cysteine increased, the release of these ions became more pronounced.

L-cysteine had a significant effect on NAD^+^, NADH content and NAD^+^/NADH in S109 (Figure 2C). With increasing L-cysteine concentrations, both NAD^+^ and NADH levels exhibited a trend of initial increase followed by a decline. Low L-cysteine concentration could significantly increase the content of NAD^+^ and NADH (*P* < 0.05), which were 1.3- and 1.6-fold higher than that of CFB. The results showed that low concentration of L-cysteine could promote the increase of NAD^+^ and NADH content. High L-cysteine concentration suppressed NAD^+^ and NADH accumulation. Intracellular NAD^+^ decreased by 49.2% relative to CFB, and compared with the low L-cysteine, NAD^+^ and NADH declined by 59.7 and 35.8%, respectively (*P* < 0.05). Additionally, L-cysteine markedly affected the NAD^+^/NADH ratio, which declined with increasing concentrations. A 2 g/L of L-cysteine promoted the increase of NAD^+^ and NADH contents, whereas higher concentrations inhibited their increase.

### 3.4 Effect of different low L-cysteine concentrations on MPs production

**FIGURE 3:**
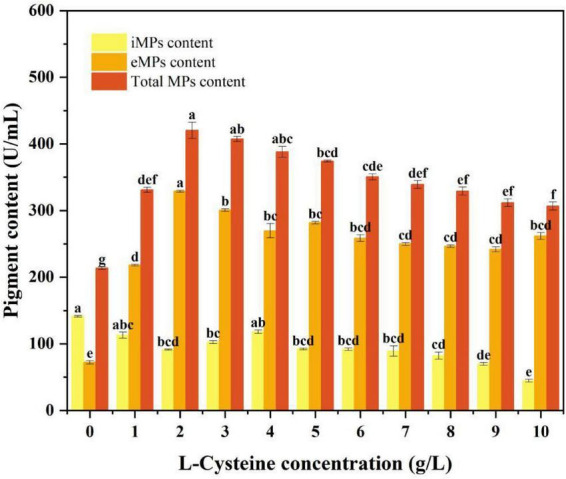
Effects of different low concentrations of L-cysteine on the ability of S109 to produce MPs. Data were presented as mean ± SD (*n* = 3). Different letters indicated significant differences among groups according to one-way ANOVA followed by Tukey’s multiple comparison test (*P* < 0.05).

The results of cell growth, eMPs production, cell membrane fluidity and permeability showed that low concentration of L-cysteine not only promoted the content of eMPs and total eMPs, but also maintained the normal growth of cells. Therefore, the optimum concentration of L-cysteine was screened and determined, and the results were shown in Figure 3.

Compared with CFB, the eMPs content increased first and then decreased after the addition of different concentrations of L-cysteine. When the concentration of L-cysteine was 2 g/L, the contents of eMPs and total eMPs were the highest. The above studies (3.1) showed that the mycelial biomass of S109 was promoted by L-cysteine. Therefore, excessive L-cysteine promoted mycelial growth, resulting in insufficient dissolved oxygen in the fermentation environment and inhibition of pigment synthesis (Zhang et al., 2023). The results once again showed that the appropriate concentration of L-cysteine promoted the synthesis of eMPs, while the excessive concentration of L-cysteine had an inhibitory effect. Therefore, 2 g/L L-cysteine was the best addition amount for high yield of eMPs, and the yield was 3.14- and 9.65-fold higher than those reported by Zhang S. et al. (2024) and Liu et al. (2020), respectively.

### 3.5 Effect of different sulfur-containing amino acids on eMP Production

**FIGURE 4:**
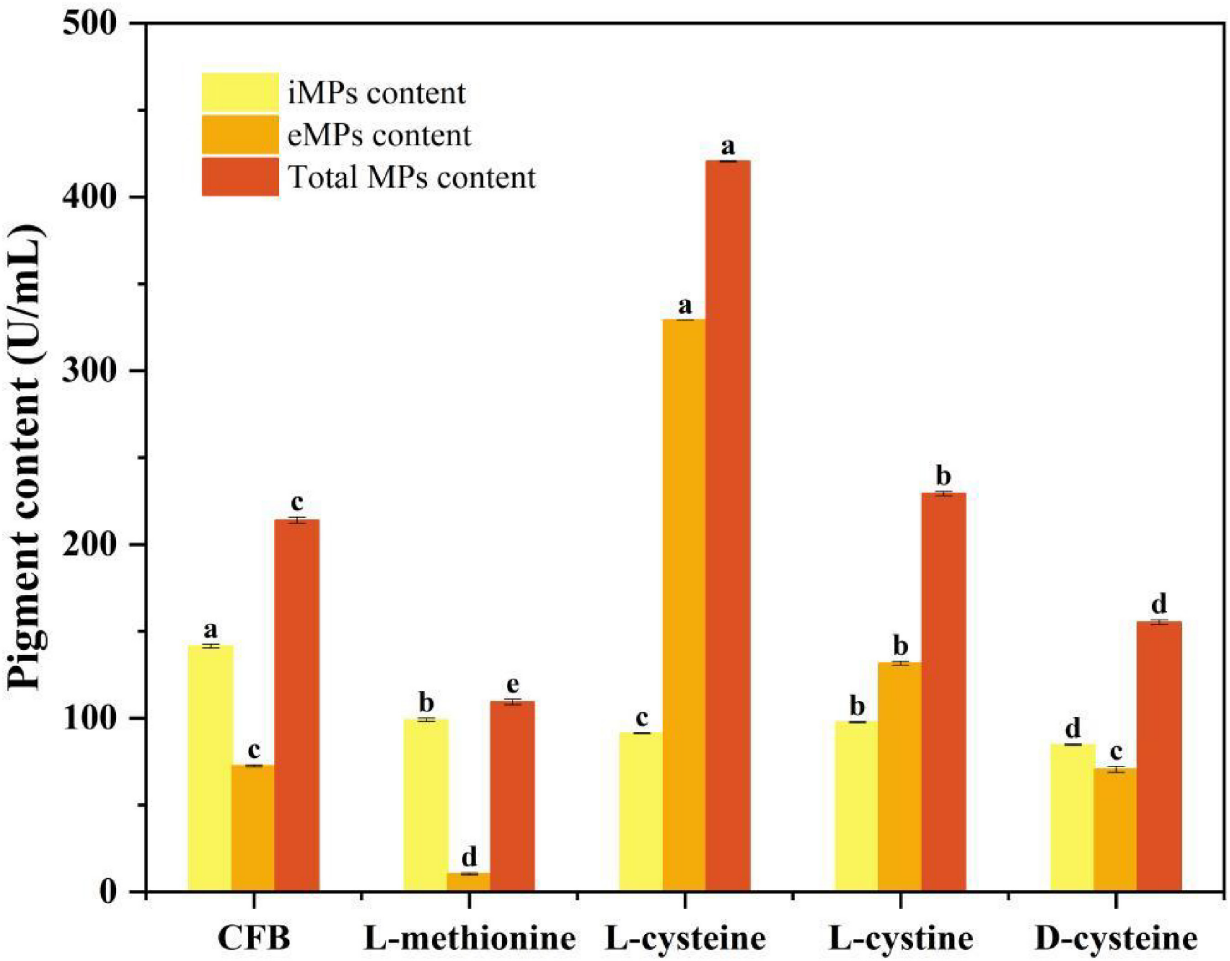
Effect of different sulfur-containing amino acids on eMP Production. Data were presented as mean ± SD (*n* = 3). Different letters indicated significant differences among groups according to one-way ANOVA followed by Tukey’s multiple comparison test (*P* < 0.05).

The effects of different sulfur-containing amino acids on eMPs production of S109 were shown in Figure 4. Compared with the eMPs yield of the CFB (72.40 U/mL), L-methionine significantly inhibited eMPs (*P* < 0.05), resulting in a yield of 10.35 U/mL. D-cysteine showed no significant promoting effect on eMPs (*P* > 0.05), with a eMPs yield of 70.56 U/mL. In contrast, both L-cysteine and L-cystine significantly enhanced eMPs, reaching 329.20 and 131.52 U/mL, respectively, with L-cysteine exhibiting the most pronounced effect (*P* < 0.05).

These results indicated that the promoting effect of L-cysteine on eMPs was not simply attributable to sulfur supplementation, since L-methionine did not enhance eMPs production and even exerted an inhibitory effect. Although L-cystine promoted eMPs synthesis, its effect was significantly weaker than that of L-cysteine (*P* < 0.05), suggesting that the presence of a free thiol group may contribute to the enhancement of eMPs. However, D-cysteine, which also contained a thiol group, did not significantly increase eMPs yield. This stereo thiol group specific difference implied that the effect of L-cysteine was associated with its participation in cellular metabolic pathways rather than a simple chemical reaction.

### 3.6 Effects of L-cysteine on the expression levels of key genes involved in MPs synthesis and secretion

As shown in the previous study (Figures 1D, 3), it was found that 2 g/L L-cysteine significantly promoted the content of eMPs in S109, and the content increased significantly in days 4–6 (*P* < 0.05). This indicated that the related genes affecting MPs synthesis and secretion have been expressed 6 days ago. Therefore, in this study, 2 g/L L-cysteine was selected to measure 16 key genes by *RT-qPCR* on days 0–5, so as to explore the effect of L-cysteine on the expression level of key genes involved in MPs synthesis and secretion of S109.

**FIGURE 5:**
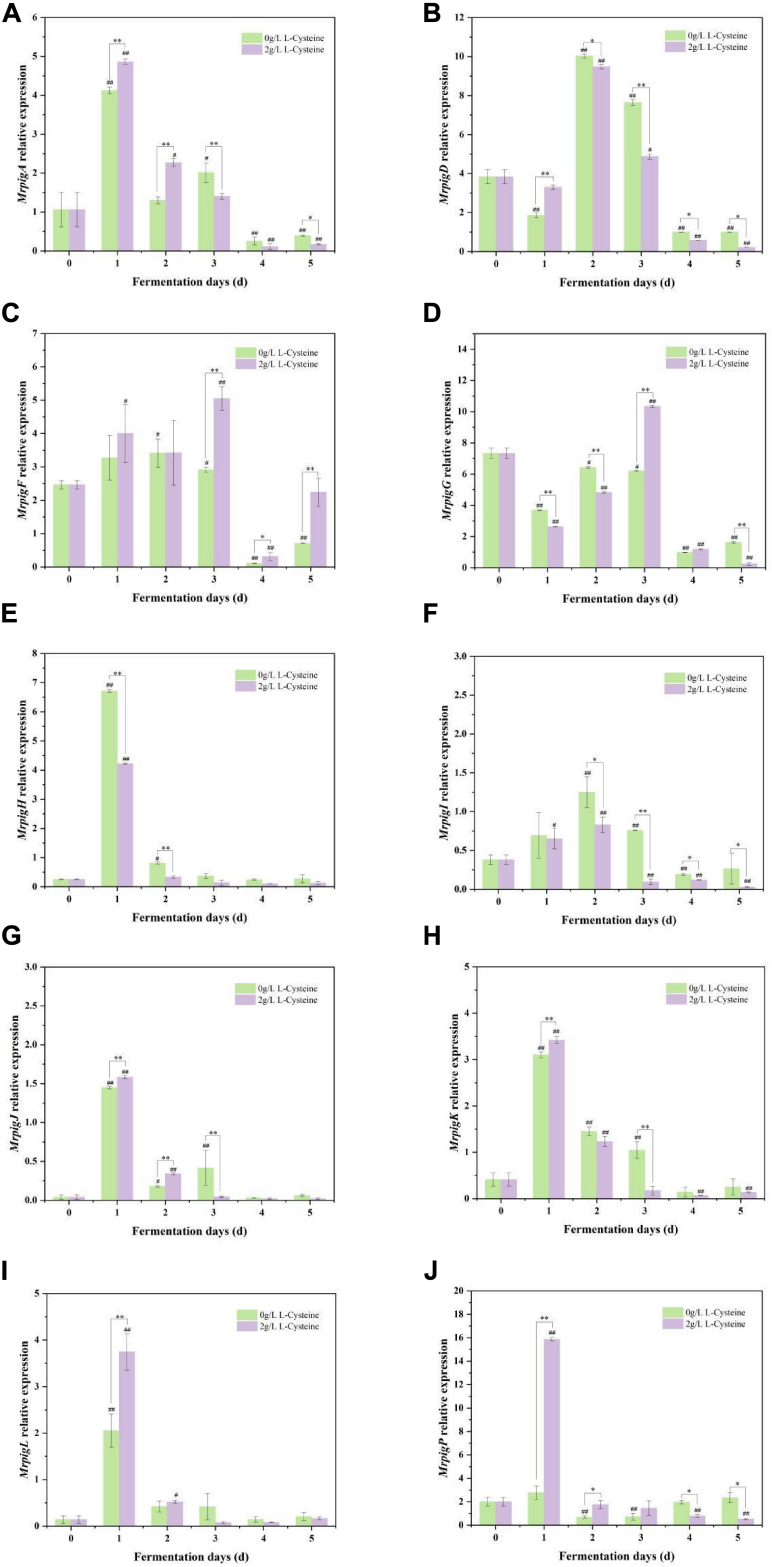
The relative expression of genes related to pigment synthesis and eMPs transport by L-cysteine. **(A–J)**, which were the gene expression of *MrpigA*, *MrpigD*, *MrpigF*, *MrpigG*, *MrpigH*, *MrpigI*, *MrpigJ*, *MrpigK*, *MrpigL* and *MrpigP*, respectively. Tukey’s multiple comparison test was used. Compared with 0 day, ^#^*P* < 0.05 or^##^*P* < 0.01 was used. Compared with 0 g/L, *P < 0.05 or **P < 0.01 was used.

*MrpigA*, *MrpigB*, *MrpigC*, *MrpigG*, *and MrpigN* were mainly involved in the PKS pathway of MPs (Figures 5A,D and Supplementary Figures 1, 2, 5). Compared to day 0, the expression levels of these genes exhibited an initial up-regulation followed by a subsequent down-regulation during both the CFB and L-cysteine-supplemented fermentation processes, indicating that core skeleton synthesis was initiated in the early fermentation phase (1–3 days) in S109. Compared with CFB, L-cysteine extremely up-regulated the expression levels of *MrpigA*, *MrpigB*, *MrpigC*, and *MrpigG* genes (*P* < 0.01). On day 1 of fermentation, the expression levels of *MrpigA* and *MrpigB* genes were up-regulated by 1- and 3-fold, respectively. By day 3, the expression levels of *MrpigC* and *MrpigG* genes were up-regulated by 2.1- and 1.8-fold, respectively.

*MrpigD*, *MrpigJ*, and *MrpigK* were primarily involved in the FAS pathway. Compared with day 0 (Figures 5B,G,H), the expression levels of *MrpigJ* and *MrpigK* were significantly up-regulated on day 1 under CFB and L-cysteine supplemented fermentation conditions (*P* < 0.01), and followed by a marked down-regulation days 3–5 (*P* < 0.01), indicating that S109 began to synthesize FAS on day 1 of fermentation. Compared with CFB, L-cysteine extremely up-regulated the expression levels of *MrpigD*, *MrpigJ*, and *MrpigK* genes on day 1 of fermentation (*P* < 0.01), and extremely significantly down-regulated the expression levels of *MrpigD*, *MrpigJ*, and *MrpigK* genes on days 3–5 of fermentation (*P* < 0.01). This indicated that L-cysteine promoted the synthesis of MPs in the early stage of fermentation (1–2 days), and down-regulated the expression of *MrpigD*, *MrpigJ*, and *MrpigK* genes in the middle stage of fermentation (3–5 days), resulting in a decrease in the synthesis of long-chain fatty acids. The fluidity of the cell membrane was affected by long-chain fatty acids.

*MrpigE*, *MrpigM*, *MrpigO*, and *MrpigI* were involved in the synthesis of MPs precursor. Compared with 0 day (Figure 5F and Supplementary Figures 3, 4, 6), the expression levels of *MrpigE*, *MrpigM*, *MrpigO*, and *MrpigI* genes in CFB and L-cysteine fermentation were first up-regulated and then down-regulated. Compared with CFB, L-cysteine up-regulated the expression of *MrpigE*, *MrpigO*, and *MrpigM* by approximately 1-, 3-, and 4-fold, respectively, and the negative regulator of MPs *MrpigI* was significantly down-regulated by 2-fold. In addition, *MrpigF* and *MrpigH* were involved in the formation of orange and yellow MPs (Figures 5C,E). L-cysteine significantly down-regulated *MrpigH* expression on day 1 (*P* < 0.01), while extremely up-regulating *MrpigF* expression from days 3 to 5 (*P* < 0.01).

**FIGURE 6:**
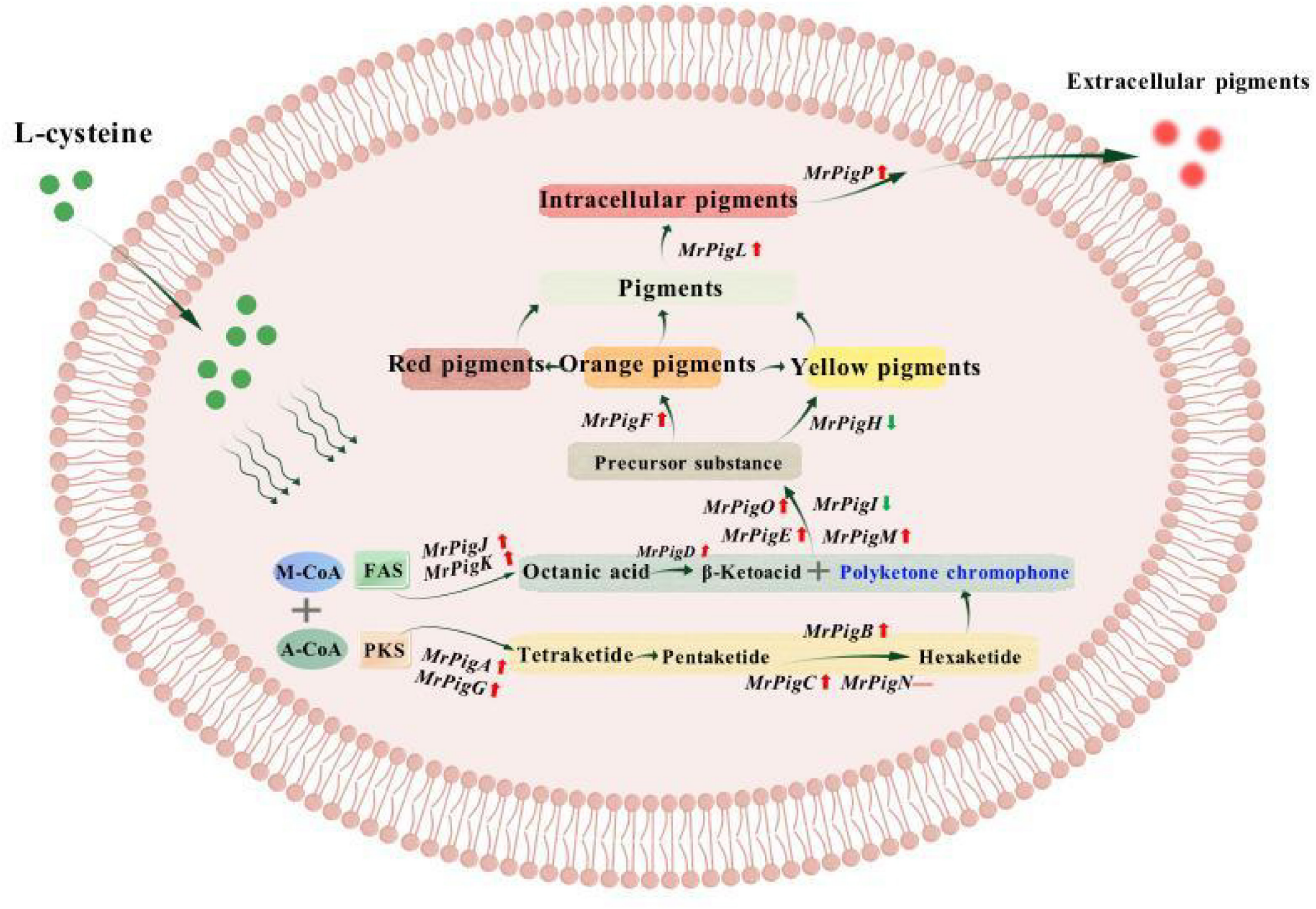
The expression levels of genes related to MPs synthesis and secretion were changed after adding L-cysteine. Polyketide synthesis—PKS, Fatty acid synthesis—FAS, Acetyl coenzyme A—A-CoA, Malonyl-CoA—M-CoA, “↓”indicated up-regulated genes, “↓”indicated down-regulated genes, “—”indicated normal genes.

*MrpigL* and *MrpigP* were primarily involved in the secretion and transmembrane transport of MPs. Compared with 0 day (Figure 5I), the expression level of *MrpigL* gene was significantly up-regulated on day 1 (*P* < 0.01), and then significantly down-regulated (*P* < 0.05). Compared with CFB, L-cysteine increased the expression of *MrpigL* gene by 1.5-fold. Compared with 0 day (Figure 5J), the expression level of *MrpigP* gene was significantly up-regulated on day 1 (*P* < 0.01). Compared with CFB, L-cysteine up-regulated the expression of *MrpigP* gene by 8-fold on day 1, and up-regulated the expression of *MrpigP* gene by 2-fold on day 2–3, and down-regulated the expression of *MrpigP* gene on days 4–5. The pathway of pigment synthesis and secretion was shown in Figure 6. L-cysteine significantly regulated the expression levels of key genes involved in the biosynthesis and secretion of MPs in S109.

## 4 Discussion

Exogenous nutrient supplementation is one of the methods that affect the synthesis and secretion of pigments by *Monascus*. This study found that different concentrations of L-cysteine have varying effects on the growth and MPs production ability of S109. High concentrations of L-cysteine promoted the growth of S109 mycelia, allowing for the full absorption of nutrients. This phenomenon leaded to an increase in the viscosity of the fermentation broth and an insufficient supply of dissolved oxygen in the fermentation environment, thereby affecting cell growth and shifting metabolism from secondary metabolites toward accumulation of lipids, amino acids, and organic acids (Khunnonkwao et al., 2024). Compared to low concentration L-cysteine, high concentration L-cysteine exhibited inhibitory effects on MPs production and pH during the later stages of fermentation, particularly after 10 days of fermentation. Excessive L-cysteine concentrations could promote the cells toward intensive biomass synthesis, accelerating carbon source depletion and shifting the metabolism toward acid production, thereby lowering the pH (Vitale et al., 2025). It might also inhibit certain deaminase activities while stimulating transamination or decarboxylation pathways, leading to the accumulation of acidic intermediates, which further contributes to the decline in pH (Farawahida et al., 2025). In summary, high concentrations of L-cysteine promoted extensive mycelial proliferation, which in turn altered the fermentation environment. This shift affected pigment metabolism and transport mechanisms, ultimately leading to the inhibition of pigment accumulation (Yin et al., 2022b). In addition, low concentration L-cysteine can not only promote the growth of S109 mycelium but also significantly increase the content of eMPs and total MPs. MPs transport was primarily driven by the concentration gradient between intracellular-extracellular environments, and exogenous additives concentration. Extensive intracellular pigment metabolism generated a concentration difference across the membrane, promoting iMPs transport. However, extracellular pigment transport was limited due to saturation inhibition by L-cysteine concentration (Chen et al., 2018). Therefore, low L-cysteine concentration conducive to the eMPs production, whereas high concentration did not increase eMPs yield and instead inhibit eMPs transport.

L-cysteine promoted the fluidity and permeability of S109 cells, as evidenced by measurements of fatty acid composition, relative conductivity, tryptophan and tyrosine fluorescence intensity, membrane potential, and ion concentration. Specifically, L-cysteine enhanced cell membrane fluidity by promoting the synthesis of unsaturated fatty acids. Relative conductivity and fluorescence intensity were significantly influenced by the concentration of L-cysteine. High concentrations of L-cysteine increased the concentration gradient across the membrane, promoting the release of intracellular substances such as iMPs, amino acids, and ions (Chen et al., 2018), thereby altering broth conductivity and increasing the cell membrane permeability. Overall, L-cysteine enhanced cell membrane fluidity, making membrane protein conformations more flexible, which facilitated iMP synthesis and transport, altered the intracellular-extracellular balance, and increased membrane permeability. In addition, high concentrations of L-cysteine inhibited the Rh123 fluorescence intensity and promoted an increase in K^+^ and Na^+^ concentrations. Change in cell membrane potential was typically associated with alterations in membrane lipid structure and fluidity, which might affect membrane permeability, while decreased in membrane potential might reflect variations in ion channel activity. Ion channel is a protein structure on the cell membrane, which is responsible for regulating the flow of ions inside and outside the cell. When the activity of ion channels changed, the flow balance of ions inside and outside the cell may be broken, resulting in variation in cell membrane potential (Chen W. et al., 2017). This implied that L-cysteine induced the opening ofq K^+^ and Na^+^ channels by altering membrane potential. At the same time, the release of K^+^ and Na^+^ would cause hyperpolarization of cell membrane potential, altering other ion channels on the cell membrane, and enhancing cell membrane permeability to promote intracellular-extracellular substances transport (Hu et al., 2024).

Fluctuations in NAD^+^ and NADH levels could profoundly impact the functionality of key metabolic pathways, including glycolysis, the tricarboxylic acid (TCA) cycle, and the mitochondrial respiratory chain (Wang et al., 2013). This study found that low concentrations of L-cysteine increased the NAD^+^ and NADH contents, while high concentrations decreased their contents. Previous studies have shown that, high concentration of exogenous compounds may alter intracellular NAD^+^ and NADH contents, thereby suppressing glycolysis, the TCA cycle, and ultimately inhibiting microbial growth (Liu Y. et al., 2021). In addition, high concentrations of L-cysteine inhibited the increase in the NAD^+/^NADH ratio. The synthesis of MPs was an energy-consuming process that required ATP as an energy source. The decrease in the NAD^+^/NADH ratio indicated an excess of NADH and a deficiency of NAD^+^, which may reduce the efficiency of oxidative phosphorylation and ATP synthesis, thereby limiting the capacity for MP synthesis. Therefore, the changes of NAD^+^, NADH content and NAD^+^/NADH ratio were affected by L-cysteine concentration. These findings showed that the concentration of L-cysteine could further affect the redox balance, energy metabolism, electron transport chain function and MPs synthesis in S109 by changing the content and ratio of NAD^+^ and NADH in S109, which was similar with Kelly et al. (2018) and Arce-Rodriguez et al. (2022). Moreover, combined with the changes of MPs content, mycelial growth, cell membrane fluidity and permeability, it could be seen that 2 g/L L-cysteine could not only significantly increase the content of eMPs and total MPs, but also properly maintain the fluidity and permeability of cell membrane and maintain the normal physiological metabolic function of cells. This result was similar with Wang et al. (2024), whereas this study further indicated its impact on membrane potential and ion channel status.

Based on the screening results of L-cysteine concentration and the comparison of different sulfur-containing amino acids, it was found that due to stereo thiol group specific differences, L-cysteine participated in cell growth and metabolism, enhanced the fluidity and permeability of the cell membrane, and promoted the synthesis of eMPs. Notably, a concentration of 2 g/L L-cysteine exhibited a significant effect. The biosynthesis of MPs mainly involves the PKS and the fatty acid synthase (FAS) pathways, where PKS constructs the pigment backbone and FAS supplies long-chain fatty acids. *MrpigA* was mainly responsible for MPs synthesis, *MrpigB* was a positive regulator of MPs synthesis, *MrpigG* was involved in the initiation of PKS pathway, and *MrpigN* was involved in the biosynthesis, transport and catalysis of secondary metabolites (He et al., 2022; Qian et al., 2021; Qin et al., 2023). Therefore, L-cysteine promoted the PKS biosynthesis pathway of MPs by up-regulating the expression levels of *MrpigA*, *MrpigB*, *MrpigC*, *MrpigG* and *MrpigN* genes. *MrpigD*, *MrpigJ*, and *MrpigK* were primarily involved in the FAS pathway, with *MrpigJ* and *MrpigK* cooperating as FAS subunits to synthesize fatty acids, while *MrpigD* played a key catalytic role and promoted the further reaction (Chen W. et al., 2017; Yu and Fischer, 2019). L-cysteine down-regulated the expression of *MrpigD*, *MrpigJ*, and *MrpigK* genes on days 2–3 of fermentation. The fluidity of the cell membrane was affected by long-chain fatty acids. The decrease in the proportion of long-chain fatty acids can increase the proportion of short-chain fatty acids or unsaturated fatty acids, thereby enhancing the fluidity of the cell membrane (Yuan et al., 2024), which was consistent with the results of the above cell membrane fluidity studies. L-cysteine up-regulated the expression of *MrpigE*, *MrpigM*, and *MrpigO* genes. Related studies have shown that knockout of *MrpigE*, *MrpigM*, *MrpigO* and other genes will inhibit the synthesis of MPs (Qin et al., 2023). These results of *MrpigF* and *MrpigH* showed that L-cysteine limited the pathway of directly generating yellow MPs from the MPs precursor, but mainly through the pathway of generating orange MPs from the MPs precursor, and then synthesizing red MPs and yellow MPs from orange MPs. *MrpigL* was mainly involved in the transport of iMPs (Liu et al., 2019b), while iMPs were mainly present in vacuoles (Chen W. et al., 2017). It was speculated that L-cysteine transported the iMPs of S109 to the vacuole by up-regulating the expression of *MrpigL* gene. *MrpigP* was responsible for encoding the major facilitator superfamily (MFS) multidrug transporter protein, which affected the extracellular secretion of MPs (Qin et al., 2023). These results speculated that L-cysteine facilitates the transport of MPs from outside the vacuole to extracellular the cell membrane by up-regulating *MrpigP* expression.

In brief, L-cysteine can significantly promote the biosynthesis of MPs in the early stage of fermentation (1–2 days). In the middle stage of fermentation (3–5 days), the fluidity of cell membrane was improved by regulating the synthesis genes of long-chain fatty acids, thereby increasing the permeability of cell membrane. Furthermore, L-cysteine significantly up-regulated the expression of MPs secretion and transmembrane-related genes, and transported iMPs to extracellular, thereby increasing eMPs. Furthermore, future research could over-express endogenous L-cysteine biosynthesis-related genes in *M. purpureus* S109 through genetic engineering methods to verify whether it can mimic the effect of exogenous L-cysteine addition, thereby constructing an engineered strain capable of autonomously and efficiently synthesizing eMPs.

## 5 Conclusion

In conclusion, the exogenous addition of low concentration L-cysteine significantly enhanced both eMPs production, and no citrinin. The underlying mechanism was attributed to the regulation of cell membrane fluidity and permeability, the change of membrane potential and ion concentration, and the increase of coenzyme content to promote the growth and metabolism of *M. purpureus* MPs, as well as the regulation of gene expression related to the synthesis and secretion of MPs, thus promoting the transport of iMPs to eMPs. This study provides an efficient, safe and low-cost method to improve the production of eMPs, and for the first time explores the regulation of L-cysteine on the production of eMPs in the fermentation process of *M. purpureus* S109, which provides an economic strategy for its application in the industrial fermentation of MPs.

## Data Availability

The datasets presented in this article are not readily available due to intellectual property considerations and confidentiality reasons. Requests to access the datasets should be directed to to Xuerui Yan, berryfood@126.com.
