# Supplementary material for: Effects of L-cysteine on the synthesis and secretion of extracellular pigments in submerged fermentation of Monascus purpureus S109
Source: Front Microbiol. 2026 Apr 1;17:1777888. doi: 10.3389/fmicb.2026.1777888 (PMC13081660; doi:10.3389/fmicb.2026.1777888)
Supplement: Supplementary file 1 [file Data_Sheet_1.docx]

**Supplementary data**

**
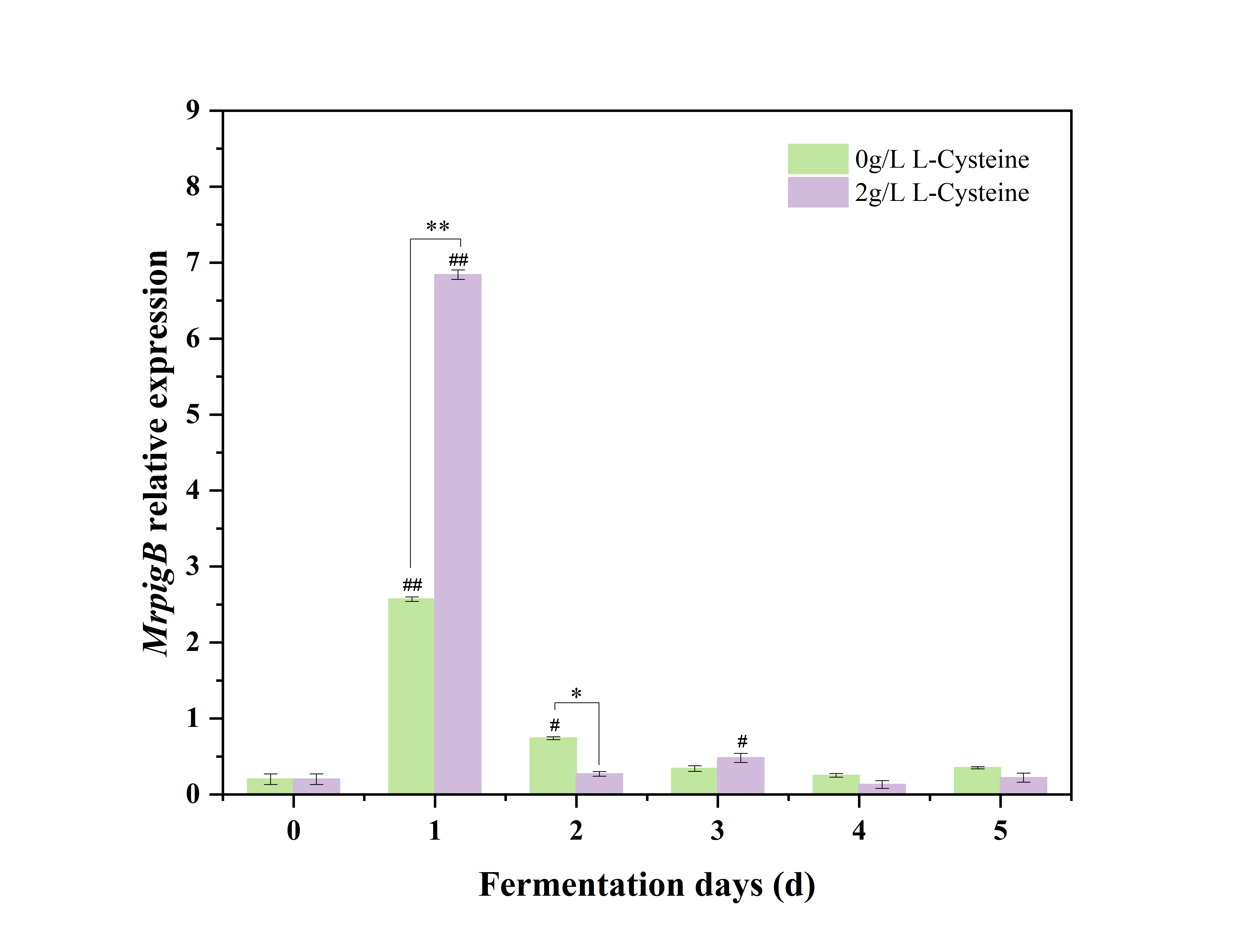
**

Fig.S1 The gene expression of *MrpigB* in *M. purpureus* S109.


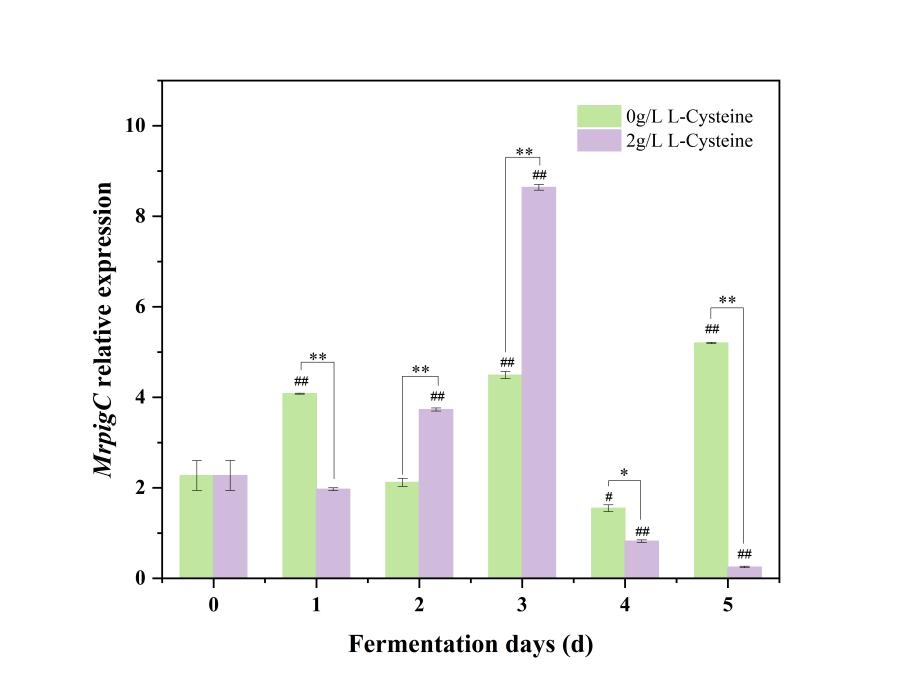


Fig.S2 The gene expression of *MrpigC* in *M. purpureus* S109.


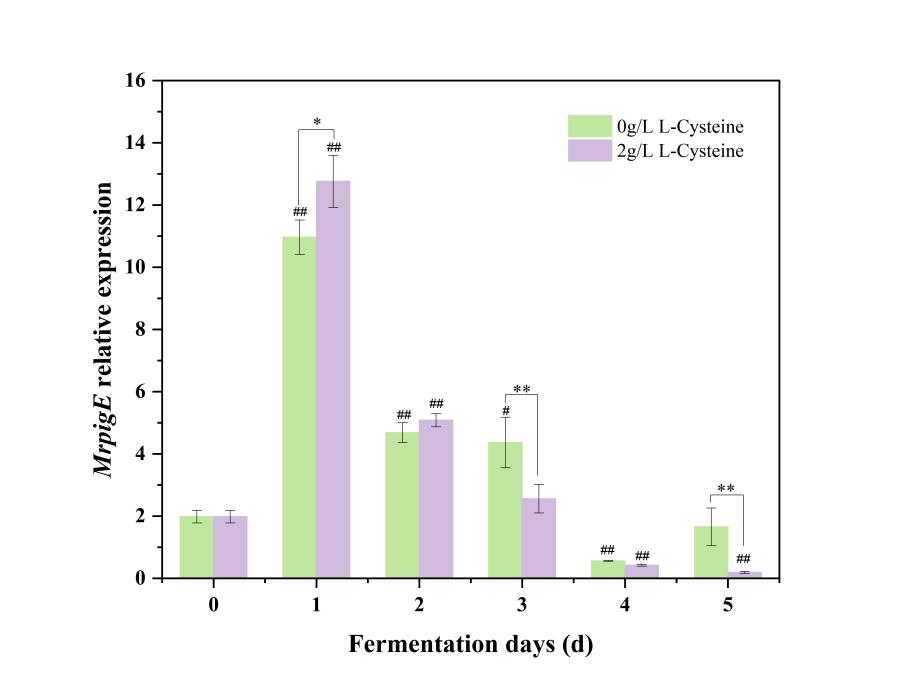


Fig.S3 The gene expression of *MrpigE* in *M. purpureus* S109.


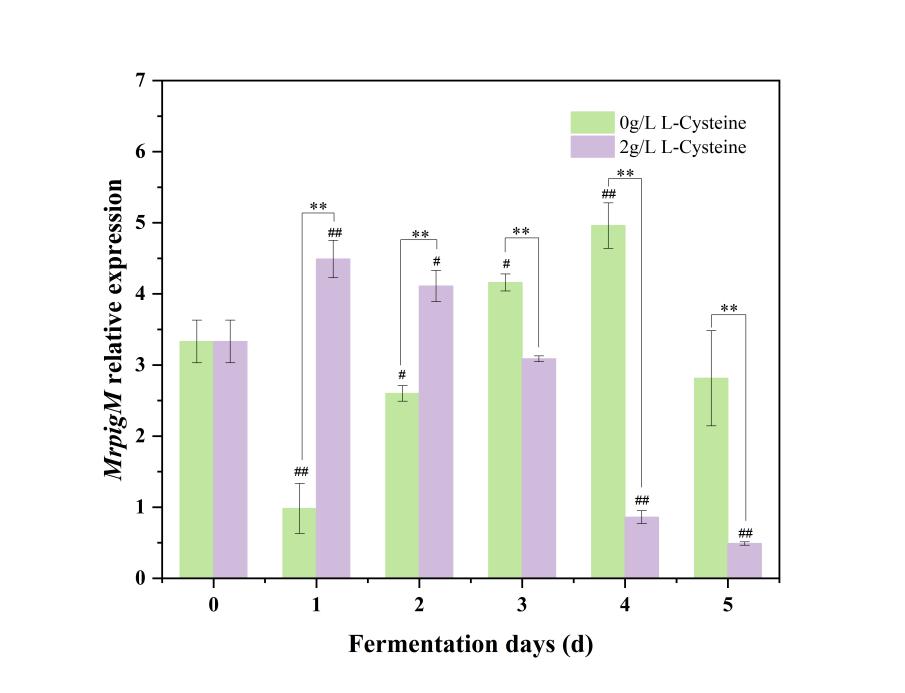


Fig.S4 The gene expression of *MrpigM* in *M. purpureus* S109.


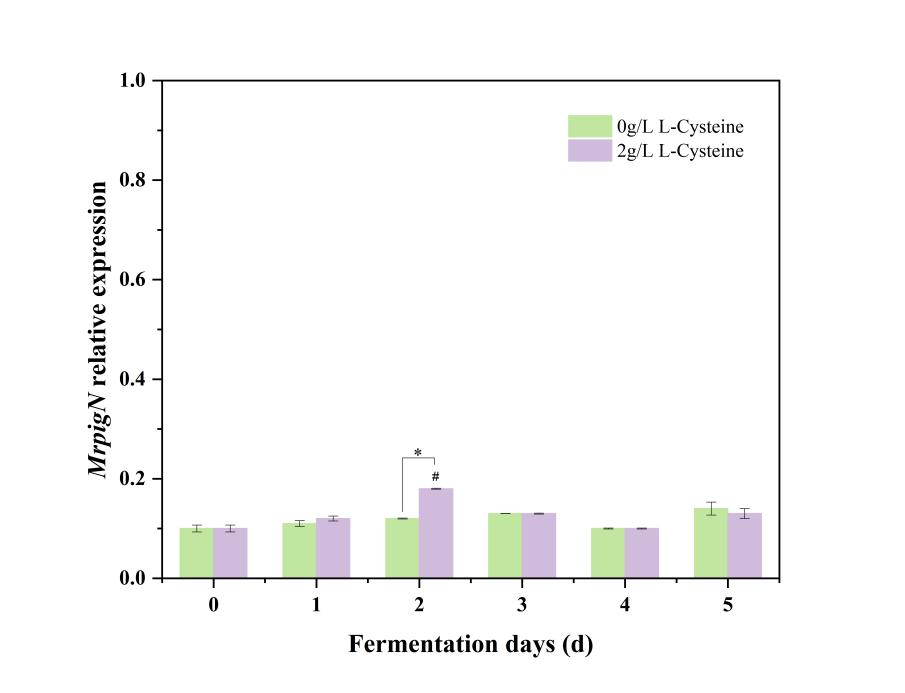


Fig.S5 The gene expression of *MrpigN* in *M. purpureus* S109.


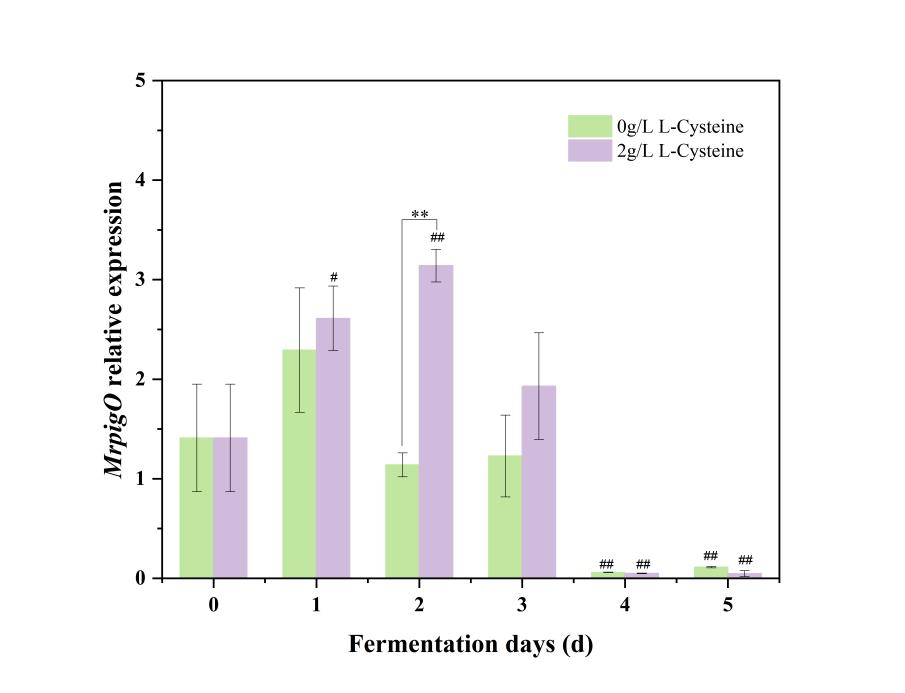


Fig.S6 The gene expression of *MrpigO* in *M. purpureus* S109.
